# Pierid Butterflies, Legume Hostplants, and Parasitoids in Urban Areas of Southern Florida

**DOI:** 10.3390/insects15020123

**Published:** 2024-02-09

**Authors:** Suzanne Koptur, Andrea Salas Primoli, Hipólito Ferreira Paulino-Neto, James Whitfield

**Affiliations:** 1 Department of Biological Sciences, International Center for Tropical Botany, Institute of the Environment, Florida International University, Miami, FL 33199, USA; asala035@fiu.edu; 2 Departamento de Ecologia, Instituto de Biociências, Universidade de São Paulo, São Paulo 05508-090, SP, Brazil; hipolito.neto@uemg.br or hipolitopaulino@gmail.com; 3 Departamento de Biociências, Universidade do Estado de Minas Gerais-UEMG/Unidade Passos, Passos 37900-004, MG, Brazil; 4 Department of Entomology, University of Illinois, Urbana-Champaign, Urbana, IL 61801, USA; jwhitfie@illinois.edu

**Keywords:** caterpillar, Diptera, exotic plants, extrafloral nectaries, Fabaceae, hostplant, Hymenoptera, native plants, parasitoid, tritrophic, urban

## Abstract

**Simple Summary:**

We collected Lepidoptera (*Phoebis* spp., Pieridae) from native and exotic legume hostplants (species of *Senna*) over several years, rearing them to test the hypothesis that caterpillars on native hostplants would be parasitized more frequently than those on exotic hostplants. Our findings were the opposite of our prediction: greater rates of parasitization occurred on Pieridae using exotic hostplants.

**Abstract:**

Are parasitoids less likely to find their Lepidoptera hosts on non-native hostplants than native hostplants? We predicted that with longer periods of coevolution between herbivores and the plants they consume, the parasitoids that provide top-down control would be more attuned to finding their hosts on native plants. To test this hypothesis, we collected immature stages of sulfur butterflies (the cloudless sulfur (*Phoebis sennae*) and the orange-barred sulfur (*Phoebis agarithe*) over a three-year period (2008–2011) from native and ornamental hostplants in the genus *Senna* in three different parts of the urban landscape of Miami, Florida, USA. We reared the immature specimens to pupation and either eclosion of adults or emergence of parasitoids and compared the levels of parasitization among the three areas, and among native vs. exotic hostplants. We found, contrary to our prediction, that caterpillars feeding on non-native leguminous hostplant species were more likely to be parasitized than those feeding on native hostplants. We discuss this surprising finding in the light of recent findings in other plant/herbivore/parasitoid systems.

## 1. Introduction

**Figure 1:**
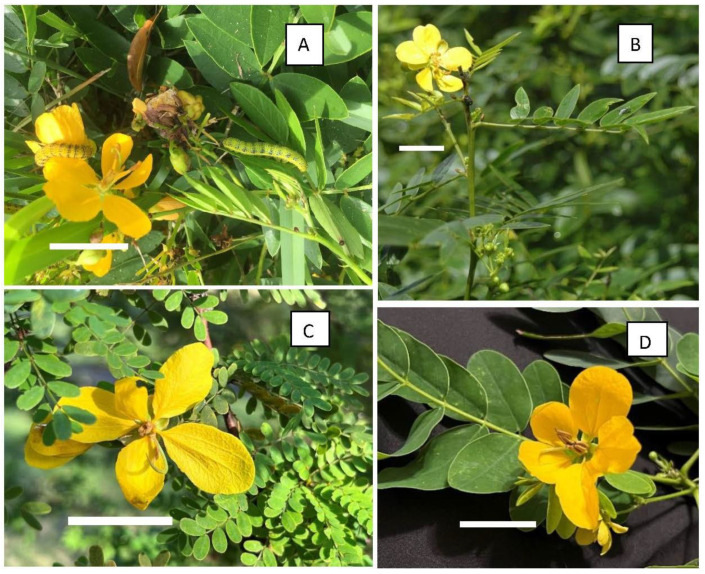
*Senna* hostplant species examined. (**A**) Native *Senna mexicana* var. *chapmanii*, with yellow caterpillar on yellow flower and green caterpillar on leaves; (**B**) native *S. ligustrina*; (**C**) non-native *S. polyphylla*; (**D**) non-native *S. surattensis.* White scale bars represent 2 cm.

The coevolution of butterflies and their host plants has led to specialization by butterflies as to which plants they seek to lay their eggs upon. The genus *Phoebis* (Lepidoptera: Pieridae: Coliadinae) is associated with Fabaceae [1], including the genera *Cassia*, *Chamaecrista*, *Lysiloma*, *Pithecellobium*, and *Senna* [2,3]. These hostplants presumably have chemical similarities (secondary plant substances) that guide their utilization by butterflies [4]. This narrow range of food plants may also be guided by morphological similarities that provide the larvae camouflage from their predators [5,6]. These legume taxa have compound leaves against which the developing caterpillars may be disguised with their color and stripes around or along their bodies, and the caterpillars match the color of what they consume: those eating leaves are green, and those eating flowers are yellow (Figure 1A) [7,8].

Some animals blend into their surroundings, evading their visually oriented predators, via a phenomenon termed “background matching” [9,10]. Caterpillars that are cryptically colored may avoid detection by staying still on the plant surface [9,11,12,13,14]. Previous work has shown *Phoebis* spp. caterpillars avoid predation by some ant species in this manner, though more aggressive ant species prod then attack them when they move [8].

Plants may receive protection from some herbivores due to their secondary chemical profile, but they may also have features such as extrafloral nectaries that attract protective agents [15,16,17], offering food for protection [18]. This potentially protective feature is well represented in the Fabaceae, notably in the genera *Chamaecrista, Lysiloma*, *Pithecellobium*, and *Senna* [16,19,20]. The extrafloral nectar produced can attract and maintain ant bodyguards but is also suitable food for other predators and parasitoids [21]. 

Though predators may account for mortality of more immature stages, parasitoids are often responsible for a sizeable proportion of deaths [22]. Parasitized hosts do not survive to reproduce themselves, and in this way extrafloral nectaries may benefit host plants by feeding adult parasitoids. Parasitoid adults are attracted to food sources such as nectar but even more to potential hosts via kairomones, chemicals produced by the interaction of the caterpillar with its food plant that alert the parasitoids to the host’s presence [23,24]. These herbivore-induced plant volatiles (HIPVs) mediate interactions among plants, their herbivores, and natural enemy communities [25].

In the urban environment, there is overall lower plant diversity than in nature, but many novel species are incorporated into the landscape as ornamentals. These are sometimes non-native species of genera found in the native flora, as is the case for the genus *Senna* in south Florida. Such situations make it possible to compare native and non-native hostplants for herbivores and their natural enemies. We predicted that the parasitoids would be more likely to find their Lepidoptera hosts on native hostplants, as they might be more familiar with the volatiles released in those interactions than those emanating from their hosts consuming non-native hostplants. We therefore sought to determine the rates of parasitism of important herbivores of *Senna* in the urban environment to test the hypothesis that *Phoebis* larvae on hostplants native to the area would be parasitized to a greater extent than those on hostplants that are exotic/non-native. 

## 2. Materials and Methods

Plants—We studied four species of *Senna* (Figure 1), all of which are woody perennials, bear yellow flowers, and have extrafloral nectaries. The two native species are *S. mexicana* var. *chapmanii* (Isely) H.S. Irwin & Barneby (referred to henceforth as *S. chapmanii*), native to pine rocklands, and *S. ligustrina* (L.) H.S. Irwin & Barneby, found in wetter habitats. The non-natives include two species widely cultivated in gardens and streetside landscapes: *S. polyphylla* (Jacq.) Irwin & Barneby and *S. surattensis* (Burm. f.) H.S. Irwin & Barneby, both of which may achieve small tree stature. *Senna polyphylla* is native to the Caribbean region, Puerto Rico and the Virgin Islands; *S. surattensis* is native to India and tropical Asia. Both have been cultivated in south Florida throughout the last century. 

Insects—*Phoebis sennae* (Linnaeus, 1758), the cloudless sulphur butterfly, is native to south Florida where they are found in flight year-round. The coloration of adults (females) and chrysalises varies seasonally. *Phoebis philea* (Linnaeus, 1763), the orange-barred sulphur, is also found year-round and has been naturalized in south Florida since 1920 [2]. The caterpillars vary in pattern and color within a species, and are quite similar, though those of *P. philea* often grow to a larger size before pupating. 

Sampling protocol—We located ten individuals of each *Senna* species studied in three areas of urban Miami-Dade County. Hostplant species occurred in landscape plantings at multiple sites, though not in equal proportions; we studied the native *Senna* spp. only in cultivation, not in natural areas. Individual host plants were at least 3 m apart from one another. We made weekly observations of these plants, examining foliage for eggs, caterpillars of all sizes (instars), and pupae on all branches that could be reached from the ground. All immature Lepidoptera were collected and transported to the laboratory, where each was reared individually in a plastic bag. We replaced their foodplants every three days, recording their development and the dates of pupation, eclosion, or death, and parasitoid presence and emergence. 

The overall urban tree canopy cover in Miami is 20%, and the three urban areas we chose (Pinecrest 25.6623° N, 80.3070° W; South Miami 25.7051° N, 80.2908° W; and Westchester/FIU 25.7548° N, 80.3273° W and 25.7562° N, 80.3755° W) differed in the amount of existing tree canopy cover and pervious surface. We compared these three areas as the greater amount of canopy indicates more vegetation and may present different environmental conditions for our study plants and the insects associated with them. 

Our collections were made over several years (2008–2011), recording the collection dates and transformations of each individual. Here, we report the number of *Phoebis* spp. herbivore eggs and immatures collected but include only those with a well-defined outcome (i.e., those that produced an adult, or parasitized) in our calculation of parasitization, not considering those that died due to a failure to thrive or other reasons (as in [26]). We considered the proportion of caterpillars that were parasitized (as in [27]), not the number of parasitoids that emerged, so that all parasitoids might be analyzed together (regardless of solitary or gregarious strategies). The numbers parasitized were compared among species (with sites combined for adequate sample sizes) using contingency table analysis; the expected numbers were derived from expectations of equal proportions parasitized among the species being compared. We then compared the parasitization of *Phoebis* spp. on the native plant species versus those on the non-native plant species using a simple chi-square test. We also compared the total numbers of adults of the two butterflies reared from each hostplant species over the course of the study. 

If the caterpillars were parasitized or died prior to pupation or eclosion, it was not possible to know which species of *Phoebis* they were [7]. When adult Lepidoptera eclosed, we determined the species and sex. While caterpillars from some other families were found less often, we do not include their data here, as the numbers were very low; we report only the Pieridae. We came to recognize morphospecies of parasitoids by their size and appearance, the shape and color of their cocoon, and from what instar caterpillar (or egg or pupa) they emerged. We later determined the identity of these parasitoids with help from entomological taxonomists. 

## 3. Results

Rates of parasitization among the species differed significantly (X^2^_3_ = 14.2, *p* < 0.005, Table 1). Rates of parasitization over all species varied over the three study areas: overall, they were highest in South Miami (17%), followed by Pinecrest (15%), and then Westchester (10%). The same patterns were reflected when considering native (11, 10, and 5%) and non-native hostplants (20, 18, and 15%) separately, though the difference in parasitization between native and non-native was significant only for Westchester (X^2^_1_ = 8.2, *p* < 0.005). Across all study areas, the natives *Senna chapmanii* and *S. ligustrina* showed only 7% of their herbivores to be parasitized overall, whereas the non-native plants showed an overall parasitization of 17%, a difference shown to be significant (X^2^_1_ = 21.2, *p* < 0.005). Contrary to our prediction, *Phoebis* spp. found and reared on native host plant species were parasitized proportionally less than those on non-native host plants.

We reared at least five different parasitoid species from eggs and larvae of *Phoebis* spp. in our study (Table 2). Unlike earlier studies in this system, we found more parasitoid species by collecting younger stages (eggs and first and second instars) of the lepidopterans. Whereas earlier we had collected later instar larvae (third instar and beyond) to allow them time to be parasitized, we did not realize then that many disappear before the third instar, dying if parasitized by parasitoids that emerge from second instar larvae or earlier. In this study, we reared only one dipteran parasitoid species from *Phoebis* spp. on only two hostplants (one native, one non-native), but four Hymenoptera, one of which was a hyperparasitoid. 

The most frequently reared hymenopteran parasitoid was the braconid wasp *Glyptapanteles cassianus* (Riley), reared from *Phoebis* on all the hostplant species (Table 2). Microgastrine braconids are normally larval endoparasitoids [24], but the subfamily also includes some egg–larval parasitoids [28], and as these small wasps emerged primarily from second-instar larvae it may be that this is the lifestyle of *G. cassianus*. As is typical of Microgastrinae [24], mature larvae of *G. cassianus* exit the host caterpillar and spin their silk cocoons on the surface of the host carcass. Though *Brasema* species are usually parasitoids of holometabolous insects living concealed in plant tissue [29,30], these were reared from free-living *Phoebis* larvae in our study. 

The one dipteran parasitoid reared in these collections, *Lespesia parviteres* (Aldrich & Webber), was much more abundant in an earlier experiment with a native *Senna* in an agricultural setting [7] along with a second undetermined tachinid species. These parasitoids are likely consumed as eggs or larvae on the foliage by caterpillars (though we do not know the mechanism of parasitization for these). We do know that they emerge from the butterfly pupal stage to pupate (or sometimes pupate in the chrysalis), producing an adult fly in a few weeks.

**Figure 2:**
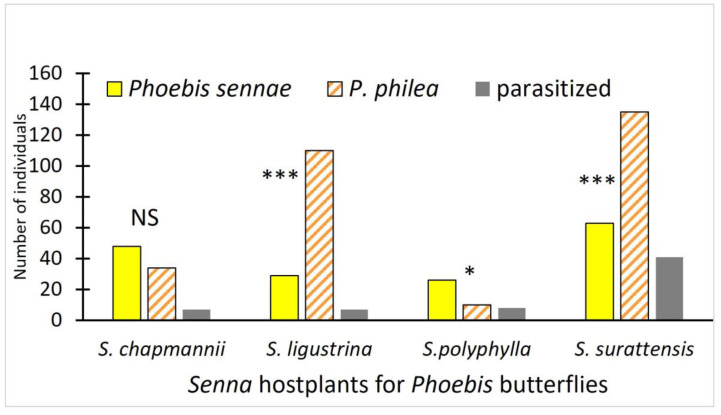
Number of adults reared on plants observed over three years in our study, and number of those parasitized. These numbers are summed over the three study areas and represent sampling 10 individuals of the *Senna* spp. on a weekly basis. Chi-sq. comparisons of numbers of the two *Phoebis* spp. on each hostplant significance indicated by stars above the pairs of bars: NS = *p* > 0.05; * *p* < 0.05; *** *p* < 0.005. The number parasitized is for both species together, as we could not distinguish species with certainty prior to adults.

More adults were reared from certain hostplants (Figure 2). *Phoebis sennae* were more successful on the exotic *Senna polyphylla* (*p* < 0.05), while *Phoebis philea* were markedly more successful on the other exotic *Senna* species (*S. surattensis*; *p* < 0.005) and on the native *S. ligustrina* (*p* < 0.005). The two butterflies did not differ significantly in number on the native *S. chapmanii* (*p* > 0.05). These numbers do not represent oviposition or host selection by the females, but rather the offspring that made it successfully to adulthood (others died of various causes, including being parasitized). 

We observed (but did not measure) that butterflies were more often seen flying and immature specimens were present on the hostplants when the plants were actively producing new leaves and flowers. Eggs were frequently deposited on flower buds and small, developing leaves. These are softer tissues that may provide nourishment easier to access for small larvae. The period of active growth is usually the months of the dry season, from November to May, but the rainfall pattern differed between the years of our study (S. Koptur and A. Salas, *personal observation*). More *Phoebis sennae* adults were reared during dry season months, while more *P. philea* were reared during the normally wetter summer months. 

## 4. Discussion

A plant is more likely to be discovered by its herbivores if it is near other plants of the same type, and herbivore density is correlated with hostplant density and patch size for some species [31] but not others [32], though other factors, such as latitude, may be even more important [33]. The species of plant upon which a caterpillar host feeds influences the extent of parasitoid attack and the diversity of the parasitoids attacking [34]. We might expect that the rate of parasitization by parasitoids on their caterpillar hosts is related to host density [35] but that is not always the case. Parasitoids are not typically monophagous but are often specialized on a repertoire (*sensu* [36]) of taxonomically or ecologically related hosts; their specificity is especially important when introducing agents for biological control of pest insects [37].

Dipteran parasitoids have been called “specialized generalists” [38], as many of them utilize a wide range of host taxa but appear specialized. Many tachinids are reared from only one or a few hosts, as their ranges are geographically restricted. The tachinid fly parasitoid reared in this study, *Lespesia parviteres*, is known from not only Pieridae hosts but other Lepidoptera families as well (including Hesperiidae, Noctuidae, and Riodinidae [39]. *Glyptapanteles* is a genus dominant in subtropical and tropical America; endoparasitic *G. cassianus* (originally described as *Apanteles cassianus* by Riley in 1881) has been reared from hosts feeding on leguminous plants in many parts of the USA. 

Non-native plants may outcompete or displace native host plants of caterpillars, and this may in turn negatively affect parasitoid populations [40,41,42]. If their host caterpillars colonize introduced plants, parasitoids may not recognize them, and caterpillars may elude parasitization [24]. Such was the logic that prompted our hypothesis that the caterpillar hosts on non-native host plants would be parasitized less than those on native host plants, but that does not appear to be the case in this study. It may be that, alternatively, the Lepidoptera hosts on native plants have had more time for natural selection to provide anti-parasitoid defenses, and that selection has acted to help hide the herbivores on native plants and that there could be no such coordination on non-native hosts. Alternatively, the chemistry of these congeners could be very similar, so that the herbivore-induced plant volatiles (HIPVs) might also be similar, so no difference in rates of parasitization would be expected.

Additionally, it may be that the HIPV chemicals are not the only thing that attracts parasitoid adults to seek herbivores on *Senna* plants. They may also seek adult nutrition in the form of nectar, produced by the extrafloral nectaries of these plants. Nectaries are present on all the *Senna* species in our study. Ants have been found to take advantage of a wider variety of legume plants with extrafloral nectaries in urban areas than in natural areas, and there is consequently a greater diversity of interactions among those plant protectors and nectaried plants than in natural areas. This is likely due to both native and non-native plants being utilized in landscaping, but only native *Senna* spp. occurring in most natural areas. It may be that parasitoid wasp and fly adults are similarly drawn to the nectar reward [43] and therefore are more likely to encounter their hosts, even on non-native plants with extrafloral nectaries. 

While ants are more influenced by the local environment, parasitoids are affected by landscape at a larger spatial scale [44]. As we have observed parasitoids at *Senna* nectaries, it is relevant that studies have found parasitoids are more numerous and diverse in urban areas with greater canopy cover and understory plant diversity [45,46] than in urban areas with less canopy cover. The differences we saw in the percentage of *Phoebis* spp. parasitized among the three areas follow this pattern. South Miami had the greatest overall percentage of parasitized Lepidoptera and is an area of Miami known for its greenery both in canopy trees and diverse yard plantings, though its existing urban tree canopy cover is less than that of Pinecrest (28.9% vs. 40%; [47]). Pinecrest, with the next largest proportion parasitized, has large properties, mostly with extensive open lawn areas, and many streets lined with canopy trees. Westchester (including the campus of Florida International University) had the lowest proportion parasitized overall and has more exposed concrete and less canopy (only 10%) than the other two areas. 

We observed more successful development of *Phoebis* spp. butterflies to adulthood on some species of hostplants. These were not uniformly native or non-native plants, but varied with each *Phoebis* sp. It will be worth examining the various factors that influence oviposition and larval development on these different legume hosts. As mentioned above, it is not only the chemical constituents of plants, but the volatiles resulting from herbivory that can affect the fate of a caterpillar. It is also important to pay closer attention to patterns of oviposition (host choice) by the butterflies, rather than just searching for immature specimens as we did in this study. Furthermore, it is essential to monitor the phenology of the hostplants, particularly the months of production of new foliage and flowers, to find out if these correlate with a greater abundance of butterflies. 

## 5. Conclusions

We found the opposite of what we initially predicted: *Phoebis* spp. were more frequently parasitized when feeding on non-native hostplants in urban areas than on native hostplants. There are undoubtedly many more things involved in plant host selection by herbivores, and attack of herbivores by parasitoids, than whether the plant is native or not. It may be that introduced plants have not evolved the same defenses as native plants as they have not experienced natural selection by local herbivores and might thus be more attractive to them; and up one trophic level, the same may be true for parasitoids attacking herbivores on those plants. Animals may evolve more fine-tuned adaptations to hosts with which they have had a long history of association, while those with shorter associations may have adaptations that are incomplete [36]. Research in other systems has shown that invasive plants evolve more constitutive but fewer inducible volatiles than native plants in response to the same herbivores [48]. Clearly, there are many more questions to answer about this tritrophic system, which may be addressed in future studies.

## Figures and Tables

**Table 1:** Number of *Phoebis* spp. juvenile stages found and reared (Found) on four species of *Senna* hostplants in three areas of Miami. The numbers of caterpillars counted (Counted)—only those that became adults or were parasitized), number and proportion parasitized (Parasitized), and totals for each species at each site are shown in columns, with overall sums for species shown in the right column, and for sites across the bottom rows, with native and non-native hostplants summed separately. An asterisk (*) indicates the plant species is not native to southern Florida. Ten shrubs/treelets of each species were monitored in each area. Different lower-case letters next to values indicate differences in column percentages (native vs. non-native plants) as determined by contingency table analysis (X^2^ values < 0.05).

Sites	Pinecrest			South Miami			Westchester			All Sites Combined $Plant Species Totals		
Plant Species	Found	Counted	Parasitized	Found	Counted	Parasitized	Found	Counted	Parasitized	Found	Counted	Parasitized
*S. chapmanii*	16	13	1 (8%)	67	55	6 (11%)	26	21	0 (0%)	109	89	7 (8%)
*S. ligustrina*	21	17	2 (12%)	1	1	0 (0%)	174	128	7 (5%)	196	146	7 (5%)
*S. polyphylla* *	5	5	2 (40%)	45	32	5 (16%)	8	7	1 (14%)	58	44	8 (18%)
*S. surattensis* *	17	16	3 (19%)	83	65	14 (22%)	231	158	24 (15%)	331	239	41 (17%)
Totals on native plants	37	30	3 (10%) a	68	56	6 (11%) a	200	149	7 (5%) a	305	235	17 (7%) a
Totals on non-native plants	22	21	5 (24%) a	128	97	19 (20%) a	239	165	25 (15%) b	389	283	49 (17%) b
Overall totals on all plants	59	51	8 (16%)	196	153	25 (16%)	439	314	32 (10%)	694	518	66 (13%)

**Table 2:** Parasitoids reared from *Phoebis* spp. (Pieridae) utilizing legume hostplants (*Senna* spp.) in urban areas of Miami, Florida. * signifies plant species is not native. X signifies parasitoid reared from caterpillars eating that hostplant species.

Taxon	Family	*Senna chapmanii*	*S. ligustrina*	*S. polyphylla* *	*S. surattensis* *
*Glyptapanteles cassianus* (Riley 1881)	Braconidae Microgastrinae	x	x	x	x
*Brasema* sp.	Chalcidoidea, Eupelmidae, Eupelminae	x	x	x	x
*Encrateola maculithorax* Ashmead, 1895	Ichneumonidae	x			x
*Lespesia parviteres* (Aldrich and Webber, 1924)	Tachinidae	x			
*Mesochorus* sp. (hyperparasitoid)	Ichneumonidae	x		x	x

## Data Availability

Upon publication, data will be made available on the FIU Dataverse at https://doi.org/10.34703/gzx1-9v95/0CBZWQ (accessed on 26 December 2023).
